# The retention characteristics of Hawley and vacuum-formed retainers with different retention protocols

**DOI:** 10.4317/jced.54511

**Published:** 2018-03-01

**Authors:** Baratali Ramazanzadeh, Farzaneh Ahrari, Zahra-Sadat Hosseini

**Affiliations:** 1 Professor of Orthodontics, Dental Research Center, School of Dentistry, Mashhad University of Medical Sciences, Mashhad, Iran; 2 Assistant Professor of Orthodontics, Dental Research Center, School of Dentistry, Mashhad University of Medical Sciences, Mashhad, Iran; 3 Assistant Professor, Department of Orthodontics, School of Dentistry, Golestan University of Medical Sciences, Gorgan, Iran

## Abstract

**Background:**

This study aimed to compare the effectiveness of two different protocols of wearing vacuum-formed retainers (VFRs) with the standard protocol of wearing Hawley retainer in maintaining the results of orthodontic treatment.

**Material and Methods:**

This single-blind randomized clinical trial consisted of 90 patients who finished orthodontic treatment at the Department of Orthodontics of Mashhad Dental School, and required removable retainers. The participants were randomly divided into 3 groups and received the following treatments. Group 1: Hawley retainers (4 months full-time and then night-only); group 2: VFR_4M (4 months full-time and then night-only); group 3: VFR_1W (1 week full-time and then night-only). The study models were prepared after debond and at 4 and 8 months later, and intercanine width, intermolar width, arch length and the Little’s irregularity index were compared between groups.

**Results:**

No significant differences were found in intercanine and intermolar widths between groups (*P*<05). Upper arch length was significantly lower in Hawley group than the two VFR groups (*p*<0.05), but lower arch length values were comparable. Upper irregularity index was significantly lower in two VFR groups compared to Hawley group (*p*<0.05), whereas in the lower jaw, only VFR_4M group showed significantly lower crowding than Hawley group (*p*<0.05).

**Conclusions:**

Both retention regimens of VFRs were more effective than Hawley retainer in maintaining arch length and tooth alignment in the upper arch. For better incisor alignment in the lower jaw, the patients should be advocated to wear VFR 4 months full-time and then at night instead of wearing Hawley retainer.

** Key words:**Essix, Hawley retainer, orthodontic treatment, retention, vacuum-formed retainer.

## Introduction

The success of orthodontic treatment mainly depends on retaining the teeth in the corrected position after the debond appointment. The term “relapse” has been defined as the regression to the original malocclusion after orthodontic correction, but it actually involves any changes in the final position of teeth following appliance removal. It is assumed that after orthodontic treatment, relapse occurs in approximately 70% of cases (1,2). Binda *et al.* (3) found that the changes following treatment are greater in females than males and are lower in adult patients. Furthermore, relapse occurs up to at least 5 years after the time of debond. Unfortunately, predicting both the occurrence and the extent of relapse is difficult in most patients (4).

 In order to counteract relapse, the retention phase has been integrated into orthodontic therapy, aiming to maintain the gained results over a long period after the debond appointment. This stage of treatment is performed by the use of fixed or removable retainers. Although the popularity of fixed retainers is increasing, but the advantages of removable retainers for both patients and orthodontists made them the most commonly prescribed appliances for maintaining treatment results.

The two commonly-used removable appliances are Hawley and vacuum-formed retainers (VFRs). Introduced in 1919, the Hawley retainer is composed of an acrylic component to which a labial bow and 2 adams clasps are attached. The labial bow passes from 4 or 6 anterior teeth and can be effective in controlling torque of incisors. The advantages of this retainer include closure of band space, closure of the extraction space (in modified types), controlling incisor torque and allowing vertical movement of posterior teeth.

Another removable retainer that has been extensively used in recent years is the vacuum-formed retainer (VFR) (5,6) commercially named as Essix. This retainer was introduced by Sheridan *et al.* (7) in 1993 and is made from polyvinyl siloxane sheets to cover all the surfaces of the teeth. The advantages of this retainer are esthetics, low cost, and simple fabrication. Breakage, occlusal wear (8) and limited vertical settling of teeth (9) are among the disadvantages of VFRs. Furthermore, this retainer is not as effective as Hawley retainer in preventing bite deepening.

There are some controversies regarding the effectiveness of Hawley and VFRs in maintaining anterior tooth alignment. Sheridan *et al.* (7) assumed that the contact pattern of Hawley retainer allows anterior teeth to move in the retention phase, whereas Essix appliance completely encapsulates the dentition and the superior part of the alveolus and thus provides better retention. However, some studies fund no significant difference in the retention characteristics of Hawley and VFRs (10,11).

There are different opinions regarding the suitable protocol for the wear of orthodontic retainers. According to Proffit *et al.* (12), Hawley retainers should be worn full-time for 3-4 months, and then night-only for at least 1 year following active treatment has ceased. In comparison, the suggested protocol for vacuum-formed retainers is all-time wear for 1 week and then night-time only (8 hours a day) for at least 1 year. Since the remodeling of periodontal fibers occurs during the first 3-4 months after appliance removal, full-time wear of VFRs for just 1 week seems to be not effective in preventing relapse in orthodontic patients.

This randomized, prospective clinical trial was conducted to assess the efficacy of Hawley and VFRs (two different retention protocols) in maintaining arch form and tooth alignment after removal of orthodontic appliances.

## Material and Methods

-Study participants

The sample of this randomized clinical trial consisted of patients who finished fixed orthodontic treatment at the Department of Orthodontics, School of Dentistry, Mashhad University of Medical Sciences, Mashhad, Iran, and were going to remove their orthodontic appliances. The included patients had the following criteria:

1- age range between 14 to 30 years

2- treated with fixed orthodontic appliances in both jaws

3- removed 4 premolar teeth for orthodontic reasons

4- presented 4-7 mm of crowding at the start of orthodontic treatment

5- tended to wear maxillary and mandibular retainers

The potential patients were excluded from the sample in the presence of the following conditions.

1- single-arch or sectional fixed orthodontic treatment

2- Hypodontia requiring tooth replacement in the retainer

3- the necessity of placing bonded retainer

4- poor periodontal condition

5- the presence of cleft lip or palate

6- orthodontic treatment combined with orthognathic surgery

7- the need for additional procedures such as interdental stripping or esthetic restorative treatment 

The sample size for each group was calculated as n=28, according to the data obtained from a previous study (10) in which the mean ± standard deviation of intercanine width in the VFR group was 26.1 ± 1.61 and that of the control group (Hawley retainer) was 27.51 ± 1.99. This gave a power of 83 per cent to detect a significant difference between group 1 and group 2 using a two-group t-test in NCSS/PASS software (NCSS Statistical Software, Kaysville, Utah), assuming an alpha significance level of 0.05. The sample size was then rounded up to 30 to allow for loss to follow up. Table 1 presents the demographic characteristics of the participants.

The enrollment started in March 2015 and was completed by December 2015. In total, 235 subjects were evaluated, but 118 did not meet the inclusion criteria and 27 were not willing to participate. Therefore, the final sample consisted of 90 eligible subjects. The study protocol was reviewed and approved by the Ethics Committee of Mashhad University of Medical Sciences and an informed consent document was taken from patients or their parents/legal guardians after a brief explanation of the treatment process.

-Interventions

At the debond appointment following the appliance removal (T1), alginate impressions were taken from the upper and lower arches and study models and working models were poured with stone plaster. The patients were then randomly allocated to one of the three retention protocols using a random numbers table. The random allocation was sealed in numbered opaque envelopes and was held by another person who was not involved in the project.

The participants in the study groups underwent the following treatments:

Group 1 (Hawley retainer): The patients in group 1 received Hawley retainer in both upper and lower jaws. This retainer consisted of an acrylic base plate, two adams clasps on first molars, and a canine to canine labial bow, all of them fabricated from 0.028-inch stainless steel wire. The labial bow was adjusted to have a light contact with the labial surfaces of the incisors. The patients were instructed to wear the retainers full-time for 4 months except for eating and oral hygiene care. After 4 months, the patients wore the retainers night-time only (for 12 hours a day).

Group 2 (VFR_4M): In this group, the participants received VFRs for upper and lower jaws. These retainers were constructed by polyvinyl siloxane sheets of 1 mm in thickness. The retainers were trimmed to provide 1-2 mm extension on labial gingiva and 3-4 mm extension on palatal gingiva. The occlusal surfaces of all the teeth were covered by the retainer including the most distally erupted tooth. The patients were instructed to wear the VFRs 4 months full-time and remove them just for eating and oral hygiene measurements. After 4 months, the patients wore the retainers night-only (for 12 hours a day).

Group 3 (VFR_1W): In this group, the patients received VFRs in upper and lower jaws. These retainers were made similar to that described in Group 2, but the wear time was different. The patients were instructed to wear VFRs 1 week full-time except for eating and oral hygiene measurements. After 1 week, the patients wore the retainers night-only (for 12 hours a day).

All retainers were made by one qualified laboratory technician. The retainers were fitted by the orthodontist within 24 to 48 hours after the debond appointment and instructions on appliance care were explained for the patients.

After 4 (T2) and 8 (T3) months of appliance removal, the patients were recalled and the alginate impressions were again taken from their upper and lower jaws. The study models were then prepared with stone plaster to assess any changes in arch dimensions and tooth alignment after 8 months of the debond appointment.

Outcome measures

The study casts were evaluated by the principal investigator to verify proper model preparation. The following measurements were made on dental models obtained at the debond appointment and at 4 and 8 months later.

1- Intercanine width (ICW): ICW was the distance between the cusp tips of right and left canines.

2- Intermolar width (IMW): IMW was defined as the distance between the mesiolingual cusp tips of the first molars.

**Figure 1:**
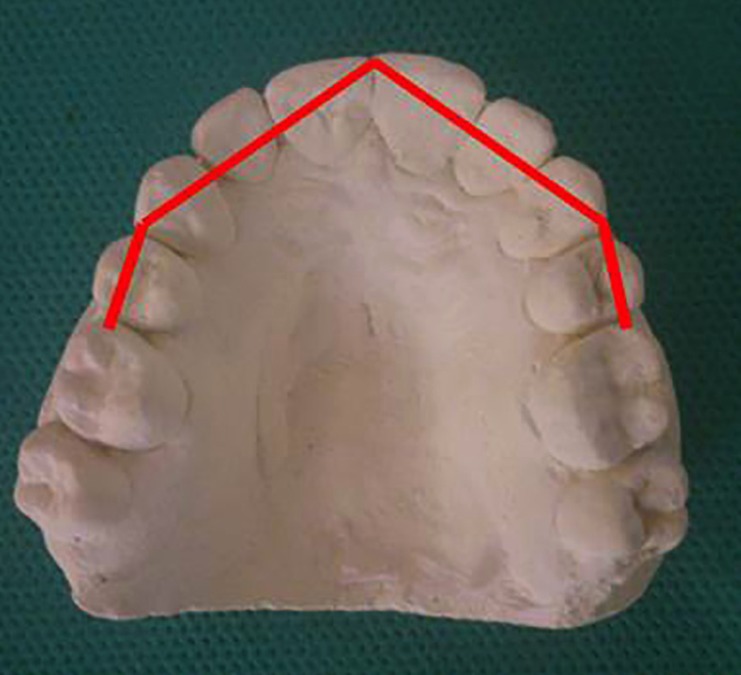
Measurement of arch length in a study model.

3- Arch length: To determine arch length, the arch was divided into 4 straight-line segments. The distance between the mesial surfaces of first molars to the distal surfaces of canines and the distance between the distal surfaces of canines to the midpoint between the central incisors were measured in both sides and summed to obtain arch length (Fig. 1).

**Figure 2:**
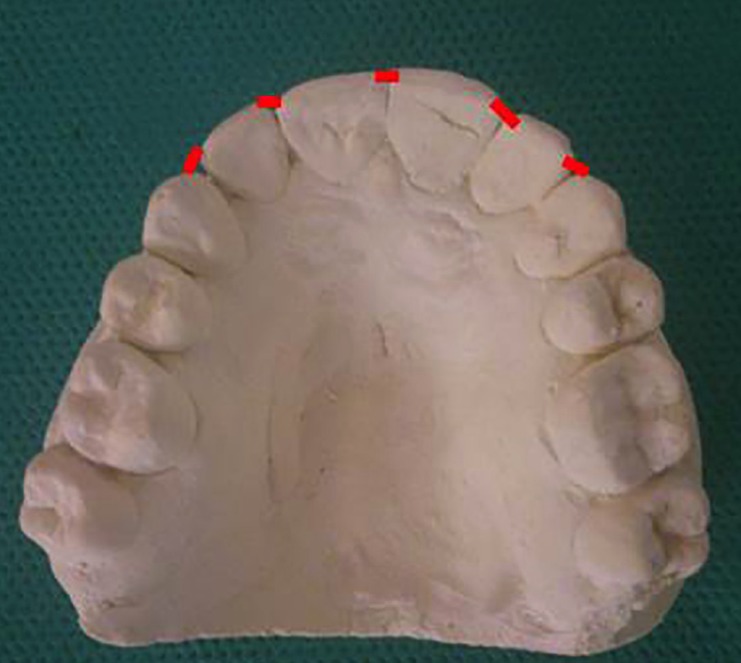
Measurement of the modified Little’s irregularity index in a study model.

4- The modified Little’s irregularity index (LII): This index was defined as the sum of the contact point displacement of incisors. To determine the LII, the most mesial and distal points on incisal edges of central and lateral incisors and the most mesial point on incisal edge of canines were marked. Then, the distances between these points were measured on neighboring teeth. By summing these 5 distances, the irregularity index was calculated for the upper and lower labial segments (Fig. 2)

All assessments were performed by a single investigator using a digital dial calipers with accuracy of 0.1 mm. A number was assigned to each study model and the models were measured randomly. The investigator who made the measurements was kept blinded to the group allocation. In order to determine intra-examiner reliability, all measurements were repeated one week later.

-Statistical analysis

The normal distribution of the data was confirmed by the Kolmogorov-Smirnov test (*P*>0.05). A paired sample t-test was used to determine the systemic error of the repeated measurements. The differences between the study groups and time intervals were compared using repeated measures analysis. T1-values were considered as covariate in the analysis to control the effect of any difference among groups at the debond appointment. When significant between-group differences were found, pairwise comparisons were made by Bonferroni test. The statistical analysis was performed using Statistical Package for Social Sciences (SPSS; version 16) and *p*-values less than 0.05 were considered statistically significant.

Results

The study included 90 patients (57 females and 33 males) in age range of 14 to 25 years. Eight patients denied from continuing the treatment, so the final sample consisted of 82 subjects. The age and gender distribution of the subjects in the study groups were comparable ( Table 1). No significant difference was found between the two measurements of the same examiner (p>0.05).

**Table 1:**
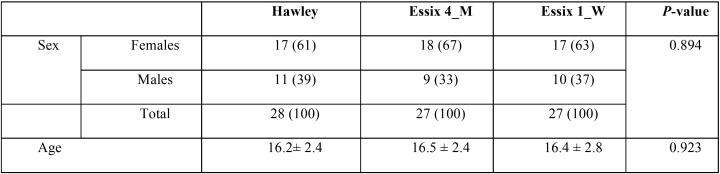
Comparison of baseline characteristics in the study groups [the quantitative variables have been shown by mean ± SD and qualitative variables by number (%)].

-Intercanine width

 Table 2 presents the mean and standard deviation (SD) of intercanine width in three groups over the period of the experiment. The repeated measures analysis revealed no significant difference in upper or lower intercanine width between the study groups (*P*>0.05;  Table 2). Intercanine width showed negligible changes over time, and the difference between 4 and 8 months of retention was not significant in any group (*P*>0.05;  Table 2).

**Table 2:**
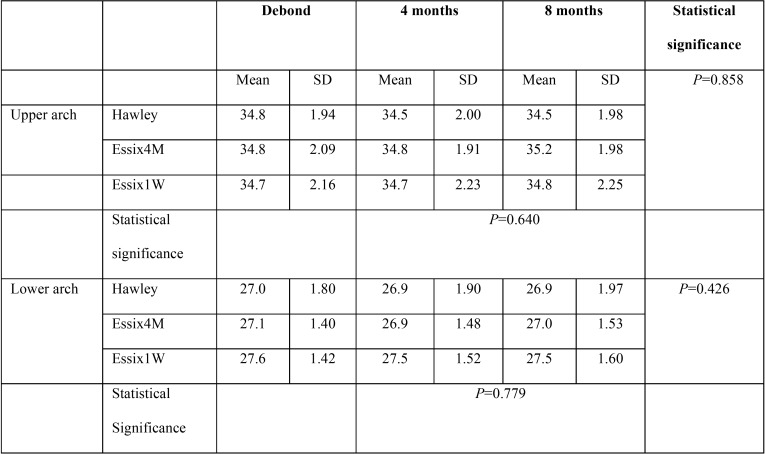
The mean and standard deviation (SD) of intercanine width in the study groups at the debond appointment and at 4 and 8 months later.

-Intermolar width

The mean and standard deviation of intermolar width in the study groups is presented in  Table 3. The repeated measures analysis revealed no significant difference either between the study groups or between the different retention intervals (*P*>0.05;  Table 3).

**Table 3:**
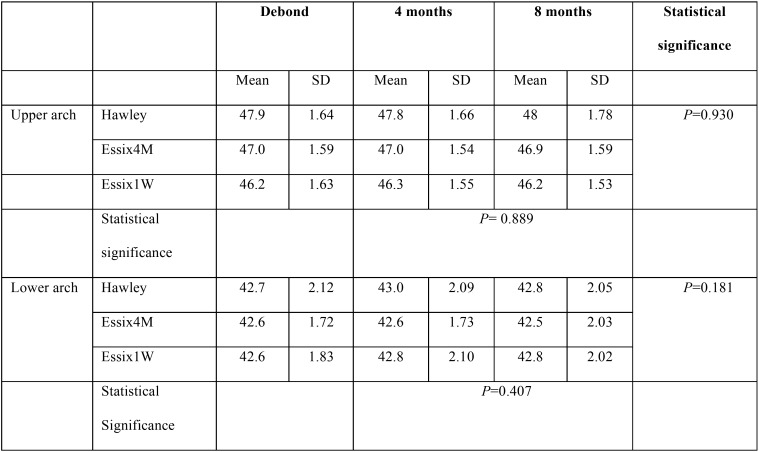
The mean and standard deviation (SD) of intermolar width in the study groups at the debond appointment and at 4 and 8 months later.

-Arch length

All groups experienced small reduction in upper and lower arch length measurements over the experiment, but the difference between 4 and 8 month values was not significant in any group (*P*>0.05;  Table 4). The repeated measures analysis revealed a significant between-group difference in upper arch length (*P*=0.007), but lower arch length values were comparable (*P*=0.680;  Table 4).

**Table 4:**
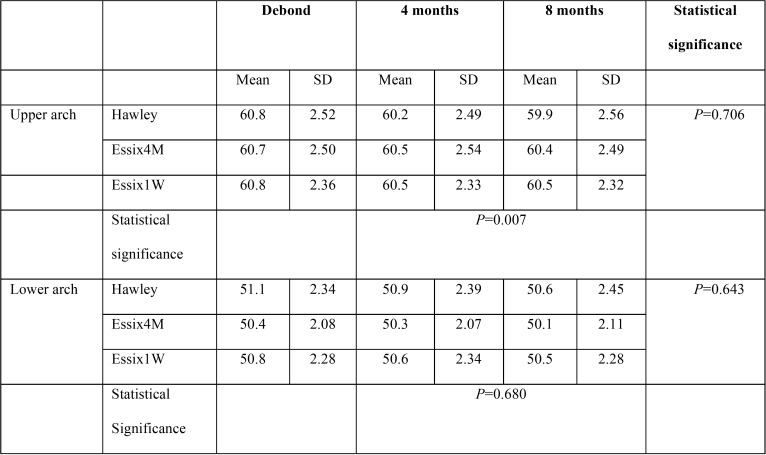
The mean and standard deviation (SD) of arch length measurements in the study groups at the debond appointment and at 4 and 8 months later.

Further analysis revealed that upper arch length was significantly lower in the Hawley group than the two VFR groups.

-The modified Little’s irregularity index

The repeated measures analysis revealed significant differences in mandibular and maxillary incisor irregularity among the three retention groups and between the different retention intervals (*P*<0.05;  Table 5). All groups showed a significant increase in upper and lower labial segment irregularity from 4 to 8 months after the debond appointment (*P*<0.05). Pairwise comparison showed that in the upper arch, the irregularity index was significantly greater in Hawley group than VFR_1W and VFR_4M groups (*P*<0.05), whereas the two VFR groups showed comparable irregularity (*P*>0.05). In the lower arch, labial segment crowding was significantly greater in Hawley than VFR_4M group (*p*=0.044), whereas other comparisons were not statistically significant (*P*>0.05).

**Table 5:**
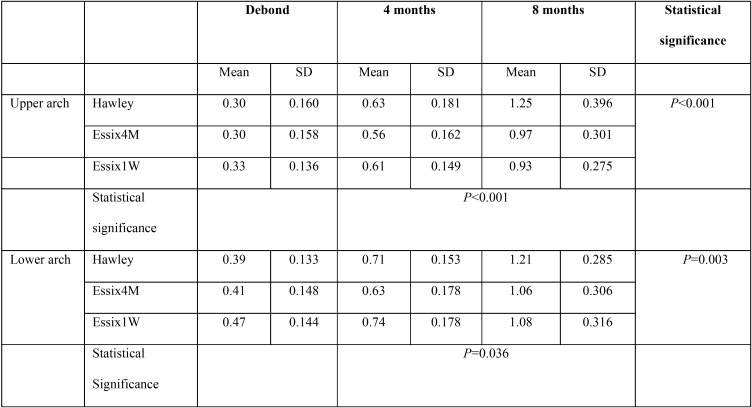
The mean and standard deviation (SD) of Little’s irregularity index in the study groups at the debond appointment and at 4 and 8 months later.

## Discussion

The present study evaluated the clinical effectiveness of Hawley and VFRs over 8 months after removal of orthodontic appliances. Ninety patients were recruited and randomized to three groups. Two retention protocols were compared for VFRs: 1 week full-time followed by night-only wear (VFR_1W) and 4 months full-time followed by night-only wear (VFR_4M). Study models were prepared at the time of debond (T1) and at 4 (T2) and 8 (T3) months later. The study took benefit from a randomized clinical design, so that confounding factors that may affect the results had the chance of being evenly distributed among the groups. Furthermore, T1-values were considered as covariate in the statistical analysis to counteract any difference between groups at the time of debond, which may affect the results over the period of the study. This was a single-blind clinical trial, because the retention appliances were different, and so the double-blind design of the study was not feasible.

In this study, both intercanine and intermolar widths showed small and insignificant changes over the study period. The alteration in intercanine width was less than 0.5 mm in the upper arch and less than 0.2 mm in the lower arch in all groups between T1 and T3 stages. The intermolar width showed less than 0.2 mm alteration over that period. Between-group differences in intercanine and intermolar widths were also insignificant in upper and lower jaws. These findings are consistent with the results of Rowland *et al.* (13), Barlin *et al.* (14), Demir *et al.* (10), Ledvinka *et al.* (15) and Kalha (16) who found that over the retention period, there was no significant difference in upper and lower intercanine/intermolar widths in patients wearing Hawley or Essix retainers. The results of this study indicate that both Hawley and Essix retainers had good performance in maintaining intercanine and intermolar widths in both arches after removal of orthodontic appliances. Although VFRs have less rigidity than Hawley retainers, they were effective in supporting transarch stability. However, in the present study the amount of relapse was assessed over 8 months after debond and so long term studies are warranted in this field.

Arch length decreased in all groups over 8 months of retention. The mean change in upper arch circumference was about 0.9 mm in Hawley group and 0.3 mm in VFR groups between the debond appointment and 8 months later. The lower arch circumference decreased about 0.5 mm in Hawley group and 0.3 mm in VFR groups through that period. Overall, Hawley group showed double the decrease in arch circumference over 8 months compared with the VFR groups. However, the difference in upper and lower arch length was not significant between the retention intervals. When arch length was compared among groups, the only significant difference was observed in the upper arch, where arch length was significantly lower in the Hawley group than the two VFR groups. Since pretreatment values were considered as covariate in the statistical analysis, the results were not influenced by between-group variations at the debond appointment. In agreement with the findings of this study, Demir *et al.* (10) reported that arch length decreased after retention in both Hawley and Essix groups, although the changes were statistically significant only in the Hawley group. It is believed that orthodontic mechanics cause increase in dental arch length during treatment, but over the retention period, arch length tends to revert to the original values (2,10,17). In contrast, Barlin *et al.* (14) found that arch length showed small decrease in both jaws with no statistically significant differences either between time periods or between the vacuum-formed and Hawley retainers.

In the present study, incisor irregularity increased in all groups over the period of the experiment. The mean change in labial segment crowding was about 0.9 mm for the Hawley group and about 0.6 mm for the VFR groups from the post-treatment (T1) to 8 months into retention (T3). The alteration in incisor irregularity between 4 and 8 months of retention was statistically significant in all groups and both arches. Rownald *et al.* (13) found that the irregularity index increased 0.56 mm in mandibular incisors and 0.25 mm in maxillary incisors from post-treatment to 6 months post-retention. Others verified that most patients experience various degrees of anterior crowding after removal of orthodontic appliances (2,17).

The outcomes of this study showed a significant difference in retention characteristics of Essix and Hawley retainers regarding the maintenance of incisor alignment. In the upper arch, incisor irregularity was significantly greater in Hawley than both VFR groups, whereas in the lower arch, crowding was significantly greater in Hawley than VFR_4M group. These findings indicate that VFRs are more effective than Hawley retainers in maintaining anterior tooth alignment in upper and lower jaws. The less increase in incisor irregularity in VFR groups could be attributed to full-contract of the retainer with tooth surfaces, whereas the point contact of Hawley retainers may allow some small tooth movements. Sheridan *et al.* (7) assumed that Essix appliance is superior over Hawley retainer in maintaining tooth alignment, because Essix retainer completely encapsulates the dentition and the superior part of the alveolus, whereas Hawley retainer has a point contact wire on the labial surface and a mass of acrylic near the lingual cervix. It is also possible that the patients comply better in wearing VFRs than Hawley retainers, because of the greater esthetics.

In the present study, there was no significant difference between the two retention protocols of wearing VFRs. Both retention regimens were more effective than the standard protocol of wearing Hawley retainer in maintaining arch form and incisor alignment in the upper jaw. However, in the lower jaw, full-time wear of VFRs for 4 months followed by night-only wear provided better efficacy in maintaining incisor alignment compared to Hawley retainer. Jaderberg *et al.* (18) compared stability after 6 months of Essix retainer using two different wear regimens: full-time wear for 3 months and thereafter at night (group A) and full-time wear for 1 week and thereafter at night (group B). They found that both retention regimens were effective in maintaining the results after orthodontic treatment and concluded that night-only wear of Essix retainer was adequate. It should be noted that patients are very perceptive to anterior dental alignment because of its strong impact on dental and smile esthetics, and therefore any small alteration in labial segment during the retention period would be disturbing for most patients.

The outcomes of this study are in agreement with those of Rowland *et al.* (13) who found that VFRs were more effective than Hawley retainer at holding the correction of the upper and lower labial segments over 6 months into retention. In contrast, Demir *et al.* (10) demonstrated that the retention characteristics of Hawley and Essix retainers were similar. Barlin et al. (14) found no statistically significant differences in a modified Little’s index of irregularity between vacuum-formed and Hawley retainers, although some degree of relapse in incisor alignment occurred in both groups over the 1 year retention period. Lindauer and Shoff (11) exhibited no significant difference between Essix and Hawley retainers regarding maintenance of incisor alignment. However, they used canine to canine retainer in VFR group and this may be the reason for the different results obtained in that study. It should be noted that the sample size, the retention protocols, and the follow-up periods were different among the studies, and these may explain the controversial results obtained in the previous trials.

According to the findings of this study, intermolar and intercanine widths cannot be considered as important variables when selecting the type of retainer for a patient. However, VFRs perform more effectively than Hawley retainers in maintaining arch length and labial segment alignment, which is very important from the esthetic standpoint for most patients. Therefore, the patients may be encouraged to wear VFRs instead of wearing Hawley retainer. For better incisor alignment in the lower jaw, the patients should wear VFRs 4 months full-time and then at night, instead of wearing Hawley retainer. Since the part-time wear of Essix retainer was more effective than the standard protocol of wearing Hawley retainer, it is also clinically acceptable to prescribe VFRs for patients who may be uncooperative in full-time wear of retention appliances. Despite the differences in clinical effectiveness between the two retainers, there are other factors that could also influence the choice of retainer such as cost, esthetics, risk of breakage, ease of fabrication and patient preference (19).

The limitations of this study were the small sample size and the short follow-up period. Since relapse occurs over long time after treatment, studies with larger sample size and longer follow-ups should be performed to compare the retention characteristics of Hawley and VFRs. Other occlusal indexes such as overjet, overbite and tooth rotation may also be assessed following the wear of different retention appliances in extraction and non-extraction cases. And the patients should be aware that despite the use of any retention appliance, relapse may still occur in some patients as a result of natural adaptation after removal of orthodontic appliances.

## Conclusions

Under the conditions of this study:

1- There were no statistically significant differences in intercanine and intermolar width between Hawley and VFR groups over 8 months of retention. Upper arch length was significantly lower in Hawley than the two VFR groups, but lower arch length was comparable among groups.

2- Both retention protocols of VFRs (4 months full-time followed by night-only wear and 1 week full-time followed by night-only wear) were more effective than Hawley retainer for maintaining the correction of upper arch length and tooth alignment. For better incisor alignment in the lower jaw, the patients should be advocated to wear VFRs full-time for 4 months and then at night-only instead of wearing Hawley retainer.

## References

[1] Melrose C, Millett DT (1998). Toward a perspective on orthodontic retention?. Am J Orthod Dentofacial Orthop.

[2] Sadowsky C, Schneider BJ, BeGole EA, Tahir E (1994). Long-term stability after orthodontic treatment: nonextraction with prolonged retention. Am J Orthod Dentofacial Orthop.

[3] Binda SK, Kuijpers-Jagtman AM, Maertens JK, van 't Hof MA (1994). A long-term cephalometric evaluation of treated Class II division 2 malocclusions. Eur J Orthod.

[4] Little RM, Riedel RA, Artun J (1988). An evaluation of changes in mandibular anterior alignment from 10 to 20 years postretention. Am J Orthod Dentofacial Orthop.

[5] Ab Rahman N, Low TF, Idris NS (2016). A survey on retention practice among orthodontists in Malaysia. Korean J Orthod.

[6] Meade MJ, Millett D (2013). Retention protocols and use of vacuum-formed retainers among specialist orthodontists. J Orthod.

[7] Sheridan JJ, LeDoux W, McMinn R (1993). Essix retainers: fabrication and supervision for permanent retention. J Clin Orthod.

[8] Gardner GD, Dunn WJ, Taloumis L (2003). Wear comparison of thermoplastic materials used for orthodontic retainers. Am J Orthod Dentofacial Orthop.

[9] Sauget E, Covell DA Jr, Boero RP, Lieber WS (1997). Comparison of occlusal contacts with use of Hawley and clear overlay retainers. Angle Orthod.

[10] Demir A, Babacan H, Nalcaci R, Topcuoglu T (2012). Comparison of retention characteristics of Essix and Hawley retainers. Korean J Orthod.

[11] Lindauer SJ, Shoff RC (1998). Comparison of Essix and Hawley retainers. J Clin Orthod.

[12]  Proffit  WR,  Fields   Jr HW ,  Sarver  DM (2013). Contemporary Orthodontics.

[13] Rowland H, Hichens L, Williams A, Hills D, Killingback N, Ewings P (2007). The effectiveness of Hawley and vacuum-formed retainers: a single-center randomized controlled trial. Am J Orthod Dentofacial Orthop.

[14] Barlin S, Smith R, Reed R, Sandy J, Ireland AJ (2011). A retrospective randomized double-blind comparison study of the effectiveness of Hawley vs vacuum-formed retainers. Angle Orthod.

[15] Ledvinka J (2009). Vacuum-formed retainers more effective than Hawley retainers. Evid Based Dent.

[16] Kalha AS (2014). Hawley or vacuum-formed retainers following orthodontic treatment?. Evid Based Dent.

[17] Sadowsky C, Sakols EI (1982). Long-term assessment of orthodontic relapse. Am J Orthod.

[18] Jaderberg S, Feldmann I, Engstrom C (2012). Removable thermoplastic appliances as orthodontic retainers--a prospective study of different wear regimens. Eur J Orthod.

[19] Tynelius GE (2014). Orthodontic retention. Studies of retention capacity, cost-effectiveness and long-term stability. Swed Dent J.

